# Study on Corrosion Mechanism of Different Concentrations of Na_2_SO_4_ Solution on Early-Age Cast-In-Situ Concrete

**DOI:** 10.3390/ma14082018

**Published:** 2021-04-16

**Authors:** Fei Zhang, Zhiping Hu, Li Dai, Xin Wen, Rui Wang, Dan Zhang, Xin Song

**Affiliations:** 1 Department of Civil Engineering, School of Civil Engineering, Chang’an University, 75 Middle Chang’an Road, Yanta District, Xi’an 710064, China; zhangfei@yulinu.edu.cn (F.Z.); 2019128040@chd.edu.cn (L.D.); 2017028001@chd.edu.cn (X.W.); wangrui@chd.edu.cn (R.W.); 2016028004@chd.edu.cn (D.Z.); 2 Department of Civil Engineering, School of Energy Engineering, Yulin University, Yulin 719000, China; 3 Shanxi Qinhan Hengsheng New Building Materials Science and Technology Ltd., Xianyang 712000, China; song516462011@163.com

**Keywords:** internal corrosion, mechanical property, hydration heat, cast-in-situ concrete, pore structure

## Abstract

The deterioration of early-age concrete performance caused by SO_4_^2−^ internal diffusion in concrete is a critical factor of concrete durability. In this study, the mechanical properties, heat of hydration, and pore structure of early-age cast-in-situ concrete with different sodium sulfate (Na_2_SO_4_) concentrations were studied. The mechanism of SO_4_^2−^ internal corrosion was evaluated by measuring the dynamic elastic modulus, compressive strength, and heat of hydration rate. Scanning electron microscopy, energy dispersive spectroscopy, X-ray computed tomography, X-ray diffraction, thermogravimetry-derivative thermogravimetry, and differential scanning calorimetry were applied to analyze microstructural variations and complex mineral assemblages of concrete samples. The results indicated that during the hardening process of cast-in-situ concrete, Na_2_SO_4_ first promoted and then hindered the hydration rate of cement, and also hindered the early strength development of the cement. As the concentration of Na_2_SO_4_ solution increases, the corrosion products of ettringite (AFt) and gypsum (Gyp) gradually increase, causing cross cracks in the concrete. The proportion of small and medium pores first increases and then decreases, and the large pores first decrease and then increase. The mechanical properties of concrete gradually decrease and diminish the mechanical properties of the concrete (thereby accelerating the damage to the concrete).

## 1. Introduction

Sulfate ion (SO_4_^2−^) erosion is one of the main factors contributing to the rapid deterioration of concrete durability. In sulfate-rich soil, rivers, lakes, groundwater, and sea water [1], there are relatively high concentrations of SO_4_^2−^ ions [2,3,4]. When considering the durability of concrete structures in salt-rich environments, it is necessary to evaluate the corrosion of the concrete in the presence of SO_4_^2−^ ions.

Numerous studies have focused on the external sulfate attack (ESA) test and the concrete mechanical properties (e.g., compressive strength, dynamic elastic modulus) after 28 days of standard curing under sulfate attack (at room temperature, i.e., 20 ± 2 °C, and relative humidity = 95%). The degradation law and mechanism have also been studied [5,6,7], and it is believed that the hydration of concrete is essentially completed before the ESA test is carried out. It is generally believed that there are two main reasons for the decrease in durability of concrete eroded by sulfate: one is a chemical attack process; the other is a physical process. Previous research regarding concrete structures indicated that chemical corrosion was the main factor leading to concrete structural damage [8]. The study found that the durability and service life of concrete gradually decrease as a result of Na_2_SO_4_ erosion. Santhanam [9] proposed that sulfate erosion could be divided into two stages—namely, the diffusion control stage and the expansion acceleration stage. In the diffusion control stage, SO_4_^2−^ ions migrate from areas of high concentration to those of low concentration through the interconnected pores and microcracks in the concrete [10]. The SO_4_^2−^ ions interact with calcium hydroxide (Ca(OH)_2_), tricalcium aluminate (C_3_A), or aluminum-phase water in the cement base. The chemical products react to produce ettringite (AFt) and gypsum (Gyp) crystals which fill the pores and cracks in the concrete [11,12,13,14,15]. AFt and Gyp accumulate in the transition zone of the pores and at the interface, causing the compactness of the concrete to gradually increase. When there is no significant crystal pressure on the pores, it has a low impact on the mechanical properties of the components; the expansion acceleration stage primarily involves the rapid growth of the AFt and Gyp corrosion products in the pores and cracks. With increasing numbers of cracks, loose structures, and new cracks [16,17,18], the mechanical properties of the components gradually decline.

When pouring underground cast-in-situ concrete structures in a salt-rich environment, it is inevitable that salt-containing media are mixed into the fresh concrete, causing internal corrosion (i.e., internal sulfate attack; ISA) in concrete. Mielenz et al. [19] first proposed the sulfate corrosion problem on prestressed concrete. Irassar [20] studied the influence factors of cement type, cement content, water–cement ratio, fly ash content, and other factors on the expansion and strength development after sulfate ISA and ESA, and proposed the compressive strength under ISA. The lower limit of the maximum expansion value is corresponding to the initial loss. Y. Fu et al. [21] studied the expansion potential of cement mortar under different curing time and temperature environment caused by internal sulfate corrosion (ISA), and the results showed that the corrosion product of sodium sulfate is cement mortar. The main factor that produces swelling. Oliveira et al. [22] proposed the AFt expansion model of concrete expansion over time through the sodium sulfate ISA test. Yaogang Tian et al. [23] used SEM-EDS and XRD to analyze the migration and corrosion law of recycled concrete (RA) carrying sulfate corrosive medium inside concrete. The results showed that as the RA aggregates get closer, the sulfate ions diffuse into the cement mortar. When the RA aggregates get closer, the corrosion products AFt and Gyp is increasing as well. Weifeng [24] used SEM and EDS to study the microstructure of concrete with different amounts of gypsum-contaminated aggregates. The results showed that the internal cracks of concrete propagate from gypsum to paste during the process of sulfate internal erosion, causing the aggregate to move from the grout and separated from the matrix. Through the comparisons of ISA and ESA presented by the aforementioned scholars, it was determined that ISA directly introduces Na_2_SO_4_ into fresh concrete as Na^+^ and SO_4_^2−^, and directly participates in the entire concrete hydration process. To date, research on the mechanism governing internal sulfate corrosion damage is relatively rare, and there is not enough experimental data to verify the relevant theoretical models. Therefore, herein, we systematically discuss the evolution of hydration heat, compressive strength, dynamic elastic modulus, and pore changes in early-age concrete under ISA conditions, and compare the impacts of corrosive environments (ISA versus ESA) on concrete durability to improve the robustness of concrete. The service performance of concrete structures in salt-rich environments is of great significance; therefore, it is crucial to study the damage evolution of early-age concrete under the action of sodium sulfate ISA.

The purpose of this work was to study the hydration heat, mechanical properties, and microstructural evolution process of early-age concrete in the presence of various concentrations of Na_2_SO_4_ to reveal the deterioration mechanism of early-age concrete via sodium sulfate ISA. In this study, varying concentrations of Na_2_SO_4_ solutions were used, with mass ratios of 0%, 3%, 5%, and 10%. The evolution process of the heat of hydration, relative dynamic elastic modulus, and compressive strength of the Na_2_SO_4_-corroded concrete were analyzed in the early stages. X-ray computed tomography was used to examine the pore structure of concrete subjected to different concentrations of Na_2_SO_4_ after 28 days of standard curing. Scanning electron microscopy (SEM), X-ray diffraction (XRD), and thermogravimetric (TG) difference analyses were used to investigate the changes in the microstructure, corrosion products, and crack evolution of concrete with different concentrations of Na_2_SO_4_.

## 2. Experimental Program

### 2.1. Raw Materials and Sample Preparation

This study used PO42.5 Portland cement, produced by Jidong Heidelberg Jingyang Cement Co., Ltd. (Xianyang, China), which was similar to ASTM Type I in the USA [25,26,27]. The chemical composition of the cement is shown in Table 1. Coarse aggregate (i.e., coarse gravel) was processed and produced in Jingyang Quarry in Shaanxi Province, and comprised particle sizes of 2.5–5 mm and 5–10 mm, which accounted for 10% and 90% of the mass, respectively. Fine aggregate corresponded to natural river sand from Xingping, Shaanxi, and had a water content of 8% and a fineness modulus of 3.24. Water was distilled before use, and the Na_2_SO_4_ reagent (analytically pure, 99.5%) was obtained from Tianjin Beichen Fangzheng Reagent Factory (Tianjin, China).

Four types of concrete samples were poured with varying Na_2_SO_4_ contents: a control group (Q), which did not contain Na_2_SO_4_, and groups 3N, 5N, and 10N. The numbers in the latter three codes represent the proportion of Na_2_SO_4_ in the concrete, which were 5.85, 9.75, and 19.5 kg, accounting for 3%, 5%, and 10% of the water mass. The group numbers and mixing ratios of the investigated concrete samples are presented in Table 2.

Before concrete pouring, raw materials were weighed out. First, coarse aggregate, river sand, and cement were stirred in a mixer for 3 min, and then, distilled water was added, and the mixture was stirred for an additional 3 min. For the 3N, 5N, and 10N samples, the concrete was poured in different batches, with Na_2_SO_4_ powder weighed, mixed with water, and added to the mixer in three doses. Finally, the samples were constructed into 100 mm × 100 mm × 100 mm and 100 mm × 100 mm × 400 mm test blocks. The samples in the molds were placed in a controlled environment at room temperature (i.e., 23 ± 2 °C) and 95% relative humidity to cure for 24 h before being removed from the molds.

### 2.2. Compressive Strength

After curing in a standard curing room for 1, 3, 7, 14, or 28 d, samples with varying Na_2_SO_4_ content were removed, and the accumulated surface moisture was dried with a rag. An HYE-2000BS electro-hydraulic servo pressure testing machine was used to test the compressive strength of concrete samples. The average value was considered as the concrete compressive strength, and the loading speed was controlled to 5 kN/s.

### 2.3. Dynamic Modulus of Elasticity

A NELD-DTV dynamic elastic modulus tester (Beijing Naerde Instrument Equipment Co., Ltd., Beijing, China) was used to test the dynamic elastic modulus of concrete samples containing different concentrations of Na_2_SO_4_. Each sample was measured every two days. For this test, the surface moisture of the 100 mm × 100 mm × 400 mm sample was removed with a rag, and the sample mass was measured using an electronic scale with accuracy to 0.1 g. Three tests were performed for each sample, and the average value was considered the dynamic elastic modulus of the concrete. The test frequency was set to 1000–3000 Hz.

### 2.4. Heat of Hydration

According to the type of concrete, the uniform water–binder ratio of 0.485 was used, mixed with different concentrations of 0%, 3%, 5%, and 10% Na_2_SO_4_ solutions. A U.S. TAM Air thermal conductivity isothermal calorimeter (Waters Technology (Shanghai) Co., Ltd., Shanghai, China) was used to test the hydration heat release and hydration rate of cement containing different concentrations of sodium sulfate over 0–7 days. These tests were carried out in accordance with ASTM C1679-2007 [28].

### 2.5. X-Ray Computed Tomography

The pore structures of the 100 mm × 100 mm × 100 mm concrete samples with different concentrations of sodium sulfate after 28 days of standard curing were investigated using a German Siemens 64-row X-ray computer tomography scanner (Siemens AG, Munich, Germany). During the scanning process, the voltage was 120 kV, the current was 300 mA, the scanning thickness of the sample was 0.6 mm, and the interval was 0.6 mm. The AVIZO 9.0.1 software program (AVIZO 9.0.1, Field Emission Instruments, Bordeaux, France) was used to perform post-processing of the concrete scan slices.

### 2.6. SEM, EDS, and XRD

To study the internal corrosion mechanism of Na_2_SO_4_ on concrete, SEM and energy dispersive X-ray spectroscopic (EDS) analyses were carried out on the 28-day standard curing concrete sample. The SEM/EDS investigations employed a German Zeiss Sigma 300 field emission scanning electron microscope (Zeiss AG, Oberkochen, Germany) to take pictures and perform elemental analysis of the samples were magnified 20,000 times and 5000 times, respectively. The samples were obtained from smashed concrete blocks, and representative fragments were selected for the analysis. The X-ray diffractometer was equipped with Bruker D8 Advance equipment (German Brook AXS, Karlsruhe, Germany). During the experiments, the voltage was 40 kV, the scanning rate was 50 scans/min, and the minimum current was 100 mA. The powder sample analyzed by X-ray diffraction was drilled 10 mm from the surface with an electric hammer and filtered through a 0.075 mm sieve.

### 2.7. Thermal Analysis

After 28 days of standard curing, the thermogravimetry of concrete powder from samples with different concentrations of Na_2_SO_4_ was analyzed using an SDT Q600 thermogravimetric/differential heat combined thermal analyzer (TA Instruments, California, TA, USA). In this experiment, the heating rate was 10 °C/min from 30 °C to 900 °C, and the nitrogen flow rate was 50 mL/min. The powder sample was drilled 10 mm from the concrete surface with an electric hammer and filtered through a 0.075 mm sieve.

## 3. Results

### 3.1. Compressive Strength and Dynamic Modulus of Elasticity

To analyze the influence of different Na_2_SO_4_ concentrations on the compressive strength and dynamic elastic modulus of early-age concrete, these parameters were measured for concrete samples after natural curing for one day after pouring (i.e., initial values), and after the concrete samples were placed in a standard environment (temperature = 20 ± 2 °C, relative humidity = 95%) for designated amounts of time; the compressive strength values of concrete were evaluated after 3, 7, 14, and 28 days, and the dynamic elastic modulus of concrete was tested every 2 days, and the average value was reported.

#### 3.1.1. Compressive Strength

**Figure 1:**
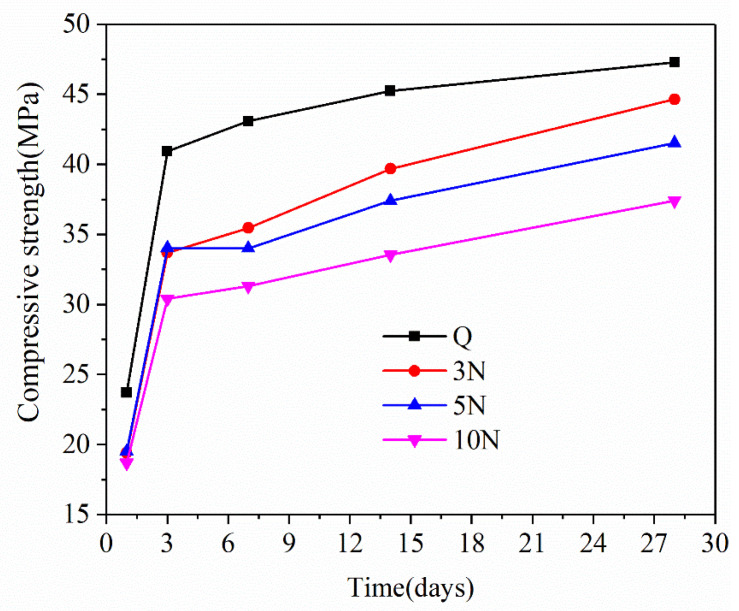
Compressive strength of concrete with different sodium sulfate content varies with time.

Figure 1 shows the changes in the compressive strength of the control group, and samples containing 3%, 5%, and 10% Na_2_SO_4_ over time. In general, the compressive strength of concrete increased gradually throughout the standard curing process. The compressive strength of the Q sample was consistently higher than those of concrete samples containing Na_2_SO_4_.

There were observable differences in the compressive strength augmentation of concrete with different curing ages and concentrations of Na_2_SO_4_. The compressive strength of concrete increased significantly in the first three days, and then continued to increase slowly thereafter. After one day of natural curing, the compressive strengths of the 3N, 5N, and 10N concrete samples were about 82.28%, 81.93%, and 78.84% of the Q sample, respectively. After standard 28-day curing, the compressive strength of the Q, 3N, 5N, and 10N concrete samples were 47.278, 44.624, 41.527, and 37.416 MPa, respectively. Therefore, the compressive strength of the 3N, 5N, and 10N concrete samples were 94.4%, 81.93%, and 78.84% of that of the Q sample, respectively. These results indicated that the presence of Na_2_SO_4_ in fresh concrete hindered the establishment of the concrete’s compressive strength. As the concentration of Na_2_SO_4_ increased, the compressive strength of the concrete gradually decreased.

The concrete’s compressive strength is mainly related to the degree of hydration, porosity, and cracks. The results showed that the addition of Na_2_SO_4_ solution into fresh concrete can inhibit the hydration rate of the cement, thereby affecting the internal skeletal structure and porosity of the concrete, and causing the relatively low strength of the Na_2_SO_4_ corrosion products. As the concentration of the Na_2_SO_4_ solution increases, the quantity of corrosion products also gradually increases, which may have a more substantial impact on the distribution of concrete pores and cracks.

#### 3.1.2. Dynamic Modulus of Elasticity

Changes in the dynamic elastic modulus can reflect changes in the concrete’s integrity, compactness, and hydration degree to a certain extent. The propagation of microcracks in the concrete and the associated reduction in compactness effectively decrease the resonance frequency, i.e., decrease the dynamic elastic modulus. The relative changes in the dynamic elastic modulus of concrete samples containing different concentrations of Na_2_SO_4_ can be calculated using Equation (1).

(1) $$\Delta E_{n}=\frac{E_{n}}{E_{0}}$$

where Δ*E*_n_ is the change in the concrete’s dynamic elastic modulus after *n* days of standard curing, *E*_0_ is the dynamic elastic modulus of concrete one day after pouring (MPa), and *E*_n_ is the dynamic elastic modulus of concrete after *n* days of standard curing (MPa).

**Figure 2:**
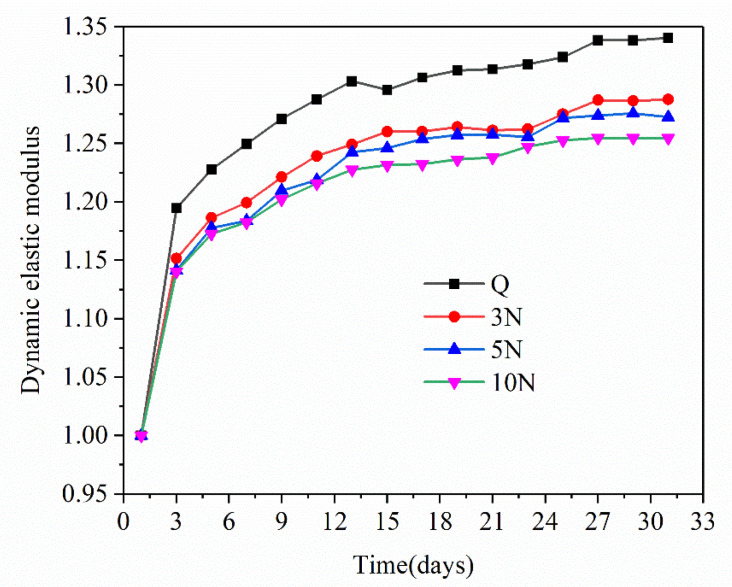
Change of dynamic elastic modulus of concrete with different sodium sulfate content with time.

It is clear from Figure 2 that as the curing time increased, the relative dynamic elastic modulus of the concrete increased rapidly (from 1–3 days) and then slowly (after day three), and that of the Q sample was generally higher than that of the concrete samples containing Na_2_SO_4_. The relative dynamic elastic moduli of the Q, 3N, 5N, and 10N concrete samples after 28 days were 1.341, 1.287, 1.272, and 1.254 times that of the samples after natural curing for 24 h, respectively. These results indicate that as the concentration of Na_2_SO_4_ in concrete increases, the relative dynamic elastic modulus of the concrete gradually decreases.

In general, the relative dynamic elastic modulus and compressive strength of concrete samples containing different concentrations of Na_2_SO_4_ are basically the same. This verified that as the concentration of Na_2_SO_4_ solution increases, the impact of concrete density, hydration rate, porosity, and cracks are more significant.

### 3.2. Heat of Hydration

**Figure 3:**
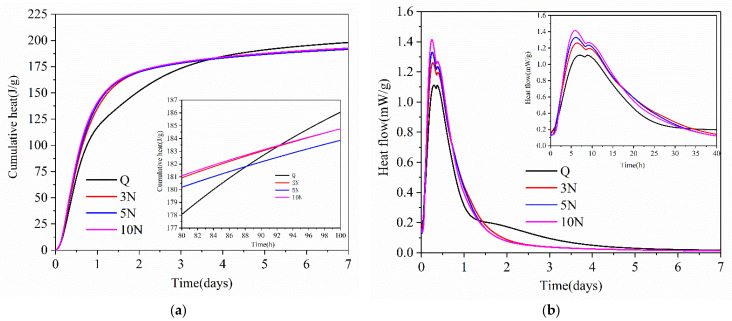
Effect of hydration heat of different content of Na_2_SO_4_ cementitious material (**a**) is the heat release curve of hydration; (**b**) is the heat release rate of hydration.

Figure 3 shows the evolution of the cement hydration heat for the control, 3%, 5%, and 10% Na_2_SO_4_ concrete samples. Based on Figure 3a, as the hydration time was extended, the heat of cement hydration first increased rapidly and then slowly. Before about 90 h, the heats of hydration of Na_2_SO_4_-containing samples were higher than that of Q sample, but after about 90 h, they were all lower than that of the Q sample. Up to two days, as the concentration of Na_2_SO_4_ increased, the heat of hydration gradually increased, but after two days, the heats of hydration of samples with different concentrations of Na_2_SO_4_ were basically the same and lower than that of the Q sample.

Based on Figure 3b, the heat flow occurred primarily in the first four days. The rate of hydration heat release underwent five stages: pre-induction, induction, acceleration, deceleration, and stabilization [29]. The reaction time in the pre-induction was too fast, and is not shown in Figure 3b. Before about 35 h, the heat release rate of the Q sample was lower than that of the samples containing Na_2_SO_4_. The first peak corresponding to the heat release rate from the Q, 3N, 5N, and 10N samples corresponded to 1.112, 1.261, 1.33, and 1.41 mW/g, and occurred at 6.99, 6.36, 6.075, and 5.925 h, respectively. The peak heat release rate of 3N, 5N, and 10N cements occurred earlier than that of the Q sample by 1.072, 0.922, and 0.636 h, respectively. As the concentration of Na_2_SO_4_ increased, the peak of the hydration heat exotherm gradually increased and tended to occur later.

Subsequently, cementitious materials containing different concentrations of Na_2_SO_4_ entered the deceleration period, during which, the hydration heat release of different concentrations of Na_2_SO_4_ decreased at different rates. At about 16 h, the hydration heat release rate of the 10N sample was larger, and that of the 3N sample became larger between 16 h and 35 h, indicating that the Na_2_SO_4_ concentrations and hydration time before the deceleration period impacted the cement hydration. The heat rate also had a certain effect. After about 35 h, the heat release rate of the Q sample was higher than that of cement mixed with Na_2_SO_4_. These results indicate that before the acceleration period, Na_2_SO_4_ can promote cement hydration, and as the concentration of Na_2_SO_4_ increases, the hydration heat release rate of cement is faster. In the late deceleration period and stable period, Na_2_SO_4_ content inhibited cement hydration. Therefore, in the standard curing environment of different concentrations of Na_2_SO_4_ concrete, the Na_2_SO_4_ solution inhibits the hydration rate of the cement. As the concentration of Na_2_SO_4_ increases, the compressive strength and dynamic elastic modulus of the concrete gradually decrease.

### 3.3. Microstructure Analyses

#### 3.3.1. XRD, SEM, and EDS Results

**Figure 4:**
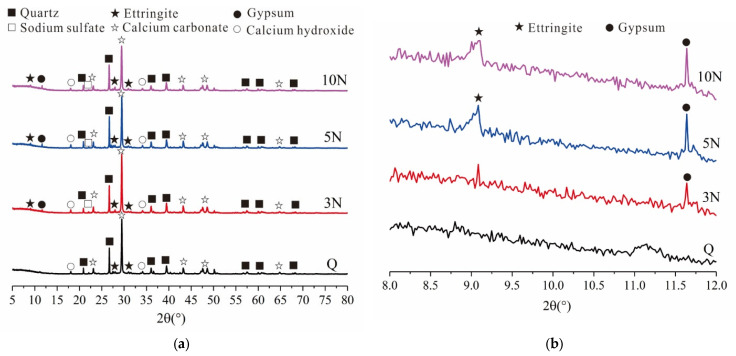
XRD diffractograms of concrete specimens (**a**): XRD of different concentrations of sodium sulfate on day 28 of concrete; (**b**): the first peaks of AFt and gypsum.

**Figure 5:**
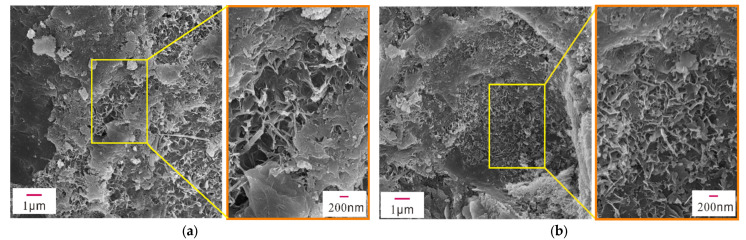
SEM images of sample Q. (**a**) Partial SEM image of sample Q, (**b**) Partial SEM image of sample Q.

**Figure 6:**
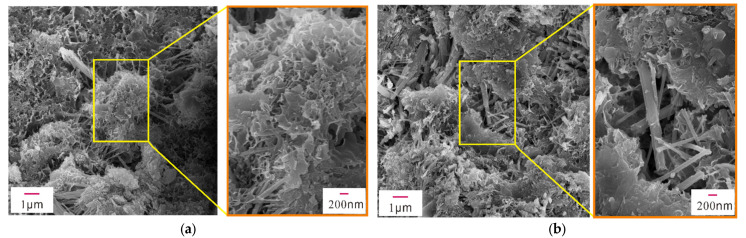
SEM images of sample 3N. (**a**) Partial SEM image of sample 3N, (**b**) Partial SEM image of sample 3N.

**Figure 7:**
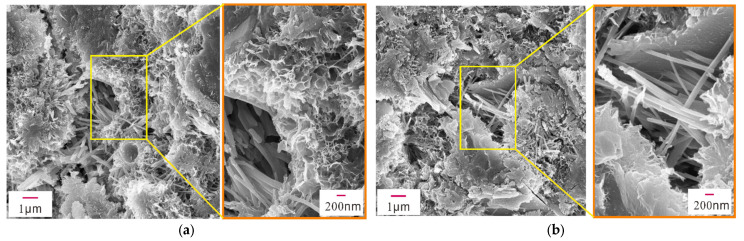
SEM images of sample 5N. (**a**) Partial SEM image of sample 5N, (**b**) Partial SEM image of sample 5N.

**Figure 8:**
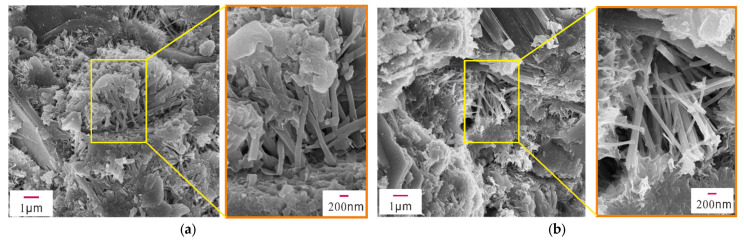
SEM images of sample 10N. (**a**) Partial SEM image of sample 10N, (**b**) Partial SEM image of sample 10N.

The mechanical properties of early-age concrete are related to the hydration products, mineral composition, pores, and cracks in the concrete. To examine their microstructures, XRD, SEM, and EDS analyses were performed on concrete samples with different concentrations of Na_2_SO_4_ (0%, 3%, 5%, 10%) after 28 days of standard curing. Figure 4 shows the XRD patterns of concrete samples containing different concentrations of Na_2_SO_4_, and Figure 5, Figure 6, Figure 7 and Figure 8 present the microstructure and composition of those samples. The analysis of the mineral composition and microstructure of concrete samples with different concentrations of Na_2_SO_4_ confirmed that the main corrosion products were AFt, Gyp, and sodium hydrate. The main corrosion reactions caused by Na_2_SO_4_ are shown in Equations (2)–(7) [30,31,32,33,34]

(2) $${\mathrm{SO}}_{4}{}^{2-}+2{\mathrm{Na}}^{2+}+10H_{2}O\to {\mathrm{Na}}_{2}{\mathrm{SO}}_{4}\cdot 10H_{2}O$$

(3) $$2\left(2\mathrm{CaO}\cdot {\mathrm{SiO}}_{2}\right)+4H_{2}O\to 3\mathrm{CaO}\cdot {\mathrm{Al}}_{2}O_{3}\cdot 6H_{2}O+\mathrm{Ca}{\left(\mathrm{OH}\right)}_{2}$$

(4) $$3\mathrm{CaO}\cdot {\mathrm{Al}}_{2}O_{3}+6H_{2}O\to 3\mathrm{CaO}\cdot {\mathrm{Al}}_{2}O_{3}\cdot 6H_{2}O$$

(5) $${\mathrm{SO}}_{4}{}^{2-}+\mathrm{Ca}{\left(\mathrm{OH}\right)}_{2}+2H_{2}O\to {\mathrm{CaSO}}_{4}\cdot 2H_{2}O+2{\mathrm{OH}}^{-}$$

(6) $$3\left({\mathrm{CaSO}}_{4}\cdot 2H_{2}O\right)+3\left(3\mathrm{CaO}\cdot {\mathrm{Al}}_{2}O_{3}\cdot 6H_{2}O\right)+26H_{2}O\to 3\mathrm{CaO}\cdot {\mathrm{Al}}_{2}O_{3}\cdot 3{\mathrm{Ca}}_{2}\mathrm{SO}4\cdot 32H_{2}O$$

(7) $$3\left({\mathrm{CaSO}}_{4}\cdot 2H_{2}O\right)+3\mathrm{CaO}\cdot {\mathrm{Al}}_{2}O_{3}\cdot 6H_{2}O+2\mathrm{Ca}{\left(\mathrm{OH}\right)}_{2}+24H_{2}O\to 3\mathrm{CaO}\cdot {\mathrm{Al}}_{2}O_{3}\cdot 3{\mathrm{Ca}}_{2}\mathrm{SO}4\cdot 32H_{2}O$$

Based on Figure 4a, as the concentration of Na_2_SO_4_ increased, the intensity of the AFt, Gyp, and Na_2_SO_4_ diffraction peaks in the concrete gradually increased, while the intensity of the Ca(OH)_2_ and C-S-H diffraction peaks gradually decreased. In part of the XRD patterns in Figure 4b, the 2θ values of the first peaks corresponding to AFt and Gyp in the concrete containing Na_2_SO_4_ were about 9.248° and 11.638°, but there was no corrosion product peak in the Q sample at the same 2θ. It was also observed that the intensity of the AFt and Gyp diffraction peaks gradually increased as the concentration of Na_2_SO_4_ increased. This indicated that as the concentration of Na_2_SO_4_ increases, the amounts of AFt and Gyp corrosion products in the concrete gradually increase, Na_2_SO_4_ hydration products appear, and Ca(OH)_2_ consumption gradually increases.

**Figure 9:**
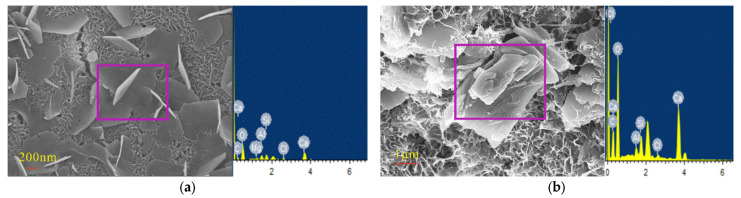

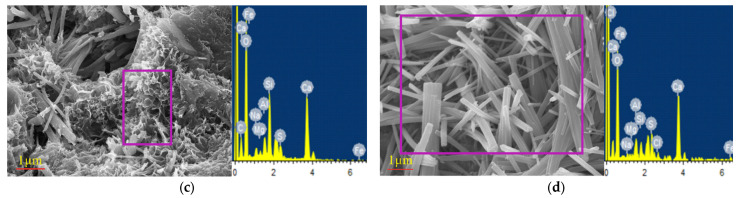
SEM images and corresponding EDS results of specimen (**a**,**b**): without sodium sulfate; (**c**,**d**): with 10% mixed sodium sulfate.

Figure 5, Figure 6, Figure 7 and Figure 8 show that the pore structure of the Q sample was covered by massive calcium carbonate or C-S-H gel structures, and there were significant amounts of flocculated cementitious material (C-S-H) and small amounts of rod-shaped AFt in the cracks. Essentially, the concrete components were combined into a whole structure. Flakey crystals were also observed, and the EDS analysis (Figure 9a) indicated that they were Ca(OH)_2_ crystals. As the concentration of Na_2_SO_4_ increased, the concrete became covered with small pores and part of the honeycomb structure. Additionally, the amount of AFt in the pores and cracks (Figure 9d) gradually increased and became denser, thus increasing its pressure on the pore wall. It was further determined that as the concentration of Na_2_SO_4_ increased, C-S-H moieties gradually changed from a block (Figure 9b) to a flocculated or honeycomb structure (Figure 9c) and became more irregular. In addition, a large number of Gyp crystals were observed in 10N.

**Figure 10:**
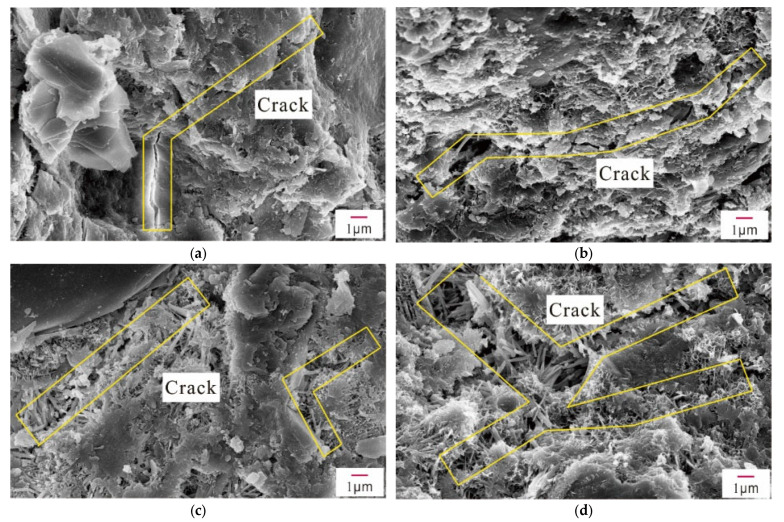
Crack evolution of concrete with different concentrations of Na_2_SO_4_ (**a**) without sodium sulfate; (**b**) with 3% mixed sodium sulfate; (**c**) with 5% mixed sodium sulfate; (**d**) with 10% mixed sodium sulfate.

Figure 10 shows shows the crack evolution induced by different concentrations of Na_2_SO_4_ in concrete samples after 28 days. Overall, there were fewer microcracks in the Q sample’s microstructure. As the concentration of Na_2_SO_4_ increased, the microcracks in the concrete gradually widened, and new cracks were generated. A significant number of rod-shaped AFt crystals accumulated in the cracks, and AFt crystals increased as the concentration of Na_2_SO_4_ increased. Similarly, interconnected cracks appeared in the 5N and 10N concrete samples.

These results showed that the corrosion products of Na_2_SO_4_ changed the microstructure of the concrete, and furthermore, that C-S-H changed from a block to a honeycomb structure with lower strength, which affected the mechanical properties of the concrete. The formation of AFt and Gyp corrosion products affected the integrity of the concrete and damaged its strength to a certain extent. It was also determined that significant amounts of AFt and Gyp accumulated in the pores and cracks in high- concentration Na_2_SO_4_ coagulation situations; furthermore, significant pressure was generated by these crystals, resulting in new and expanded cracks in the concrete. Therefore, as the concentration of Na_2_SO_4_ increased, the corrosion products also gradually increased in concentration, and the porosity gradually decreased. The deterioration rate of the mechanical properties of concrete is gradually accelerating. However, the excessive crystal stress generated by relatively high concentrations of Na_2_SO_4_ in the concrete widened the cracks and generated new cracks, resulting in reduced compactness, increased porosity and accelerated the damage of concrete mechanical properties, which further reduce the service life and durability of concrete.

#### 3.3.2. Thermal Analysis

**Figure 11:**
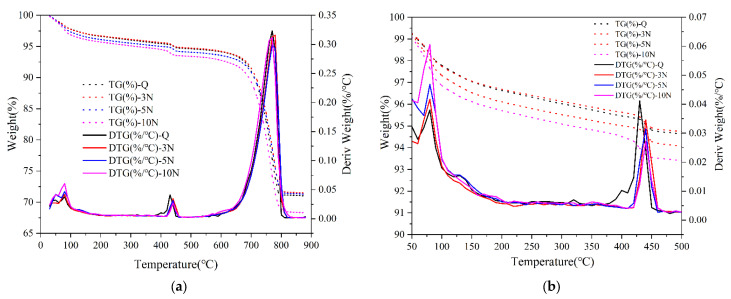
TG/DTG curves of concrete specimens (**a**) different of Na_2_SO_4_ contents; (**b**) the first peaks of AFt and Gyp.

Figure 11 shows the weight loss of corrosion products in concrete samples containing different concentrations of Na_2_SO_4_ on the 28th day. The differential TG (DTG) curves determine the temperature range corresponding to the weight loss of corrosion products. The crystalline composition of corrosion products of Na_2_SO_4_ concrete was thus verified.

It is clear from Figure 11a that the three main weight loss stages or peaks related to the corrosion products and concrete hydration products of 0%, 3%, 5%, and 10% Na_2_SO_4_ concrete appeared in TG/DTG analysis, the first weight loss stage or peak temperature was 80–105 °C [35,36,37], which was mainly resulted from the decomposition of Gyp and AFt. The second weight loss stage or peak temperature was 400–500 °C, which was primarily caused by the decomposition of Ca(OH)_2_. The final weight loss stage or peak temperature was 600–800 °C, which was due to the decomposition of calcium carbonate [36,38,39,40]. Based on Figure 11b, as the concentration of Na_2_SO_4_ increased, the absorption peak areas of Gyp and AFt gradually increased, while the absorption peak areas of Ca(OH)_2_ gradually decreased. These results confirmed that the damage to the mechanical properties of concrete is mainly caused by the corrosion products Gyp and AFt. At the same time, as the concentration of Na_2_SO_4_ increases, the production of Gyp and AFt corrosion products increased, and the Ca(OH)_2_ concentration in concrete decreased. The mechanical properties of concrete mainly show the rapid decline of compressive strength and dynamic elastic modulus.

#### 3.3.3. X-Ray Computed Tomography Results

The porosity of concrete has always been considered as the main factor governing the mechanical properties of concrete. To study the influence of different concentrations (0%, 3%, 5%, 10%) of Na_2_SO_4_ on the pore structure of concrete, X-ray computed tomography was applied for the samples cured for 28 days. Owing to the complexity and irregularity of the internal pore structure of concrete, the shape of the pores can be standardized as spheres, and the equivalent diameter of the pores can be determined by calculating the pixel volume of each sphere. Equivalent diameters <2000 μm are regarded as small pores, diameters of 2000–4000 μm are mesopores, and those ≥4000 μm are macropores.

**Figure 12:**
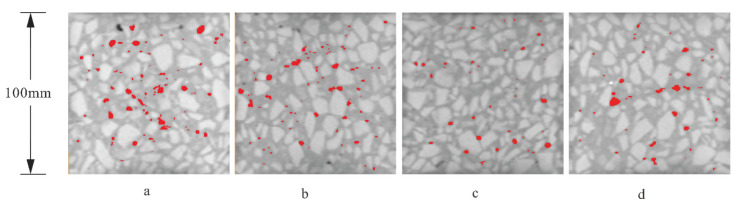
Scanning section CT images of different concentrations of Na_2_SO_4_ in concrete (**a**) without sodium sulfate; (**b**) with 3% mixed sodium sulfate; (**c**) with 5% mixed sodium sulfate; (**d**) with 10% mixed sodium sulfate.

Figure 12 shows the X-ray computed tomography slices of concrete containing different concentrations of Na_2_SO_4_; the red areas represent the pore structure. Table 3 shows that as the concentration of Na_2_SO_4_ increased, the pore volume ratio in the concrete first decreased and then increased, and the pore area in the 5N sample was the smallest. It is clear from Figure 12 that when the concentration of Na_2_SO_4_ changed from 0% to 5%, the area and density of the red area in the concrete decreased. When the Na_2_SO_4_ concentration was greater than 5%, the red area of the concrete became denser and more obvious with increasing concentrations of Na_2_SO_4_; additionally, the red pores became connected.

**Figure 13:**
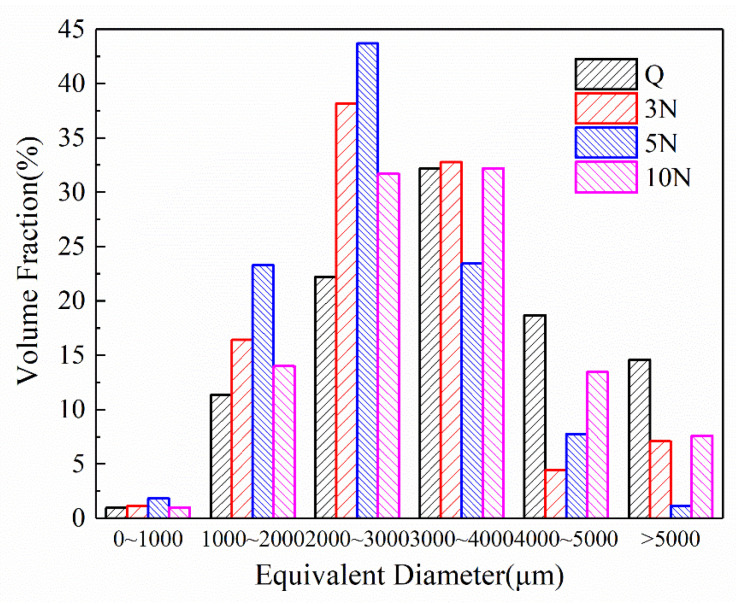
Equivalent diameter pores of concrete with different content of sodium sulfate on the 28th day.

To evaluate the effect of different concentrations of Na_2_SO_4_ on the pores of early-age concrete the pores with equivalent diameters of 0–1000, 1000–2000, 2000–3000, 3000–4000, 4000–5000, and ≥5000 μm were analyzed. A histogram depicting the relationship between the equivalent diameters of concrete pores and their Na_2_SO_4_ content is shown in Figure 13. The proportion of small and medium pores in the equivalent diameter range of 0–3000 μm increased significantly after mixing in Na_2_SO_4_. Among the concretes containing Na_2_SO_4_, the proportion of small pores in sample 5N was the largest. When the content of Na_2_SO_4_ was 10%, the proportion of small and medium pores was significantly less than that in 5N. This indicated that the reason of the low-concentration Na_2_SO_4_ concrete damage is mainly the corrosion products of gypsum and AFt. As the concentration of Na_2_SO_4_ increases, the corrosion products of Gyp and AFt in concrete gradually increase, and the proportion of small and medium pores increases significantly. Excessive concentration of Na_2_SO_4_ produces a large amount of corrosion products of Gyp and AFt in concrete, which is expansion stress generated by corrosion products much higher than the resistance of the matrix. Expansion stress causes new cracks in the concrete. At the same time, there are interconnected pores in the concrete, which reduces the small and medium pores and increases the large pores, so that the mechanical properties of concrete gradually decrease with the increase of sodium sulfate concentration [41,42,43,44], and high-concentration sodium sulfate concrete drops faster.

## 4. Discussion

In this study, tests of compressive strength, relative dynamic elastic modulus, and heat of hydration showed the damaging effects of different concentrations of Na_2_SO_4_ on early-age concrete. Analysis of XRD, SEM, EDS, TG (DTG/DSC), and X-ray computed tomography results revealed how various concentrations of Na_2_SO_4_ impacted concrete minerals, microstructures, and pores. Previous studies generally indicated that Na_2_SO_4_ is consumed in the process of cement hydration, resulting in little or no effect on the mechanical properties and microstructure of concrete [21,22,45]. At that stage, the erosion process of Na_2_SO_4_ on concrete is mainly via ESA; however, to date, there are few studies on ISA in early-age concrete.

### 4.1. Sodium Sulfate

After adding Na_2_SO_4_ solutions to fresh concrete, the Na_2_SO_4_ exists in the form of Na^+^ and SO_4_^2−^. Na_2_SO_4_ can react with Ca(OH)_2_ precipitated during cement hydration to form Gyp. This process consumes a large amount of Ca(OH)_2_, which promotes the hydration of tricalcium silicate(C_3_S) and dicalcium silicate(C_2_S) [46], and then tricalcium aluminate (C_3_A) reacts with Gyp to form a swelling product AFt. When hydrated for a certain period of time, Na_2_SO_4_ reacts with Ca(OH)_2_ to generate NaOH, which increases the OH^−^ concentration in the environment, thus increasing the alkalinity and inhibiting the continued formation of Ca(OH)_2_. Simultaneously, the unconsumed Gyp and the over-corrosion product AFt wrap the surface of the cement particles, reducing the hydration rate of the cement.

When the concentration of Na_2_SO_4_ increased, XRD results indicated that the intensity of the Gyp and AFt diffraction peaks gradually increased. This shows that the AFt and Gyp corrosion products in concrete gradually increased in concentration with greater amounts of Na_2_SO_4_. As the concentration of Na_2_SO_4_ increased, the hindrance of cement hydration became clearer in the late deceleration and stable periods. As shown in Figure 3, before the deceleration period, as the Na_2_SO_4_ concentration in the concrete increased, the cement hydration rate gradually increased. From the late deceleration period into the stable period, the cement hydration rate of the Q sample was higher than that of the Na_2_SO_4_-containing cement samples, which further verified that Na_2_SO_4_ promoted cement hydration in the early stage and inhibited the development of cement hydration later.

### 4.2. Mechanical Property

By evaluating the compressive strength and dynamic elastic modulus of concrete samples containing different concentrations of Na_2_SO_4_, it can be concluded that the overall mechanical properties of the Q sample were greater than those of concrete samples containing Na_2_SO_4_. The XRD, XRD, SEM, EDS, TG (DTG/DSC), and X-ray computed tomography analytical results showed that the addition of Na_2_SO_4_ was the main cause of damage to the mechanical properties of concrete.

The Q sample contained Gyp and AFt, mainly because of the addition of CaSO_4_ in the cement, which delayed the cement setting process. At this time, there were small amounts of Gyp and AFt in Q, which filled the pores and cracks formed by hydration, the proportion of mesopores is higher than that of large and small pores, causing no damage to the mechanical properties or microstructure of the concrete [47]. After adding Na_2_SO_4_ to the fresh concrete, it immediately participated in the corrosion reaction. In the Na_2_SO_4_ ISA, the concrete itself needs to be provided with Ca^2+^. Generally, the Ca^2+^ in concrete comes from silicate and C-S-H. In fact, C-S-H has the most direct impact on the compressive strength and elastic modulus of concrete. Therefore, the mechanical properties of concrete decrease after Na_2_SO_4_ erosion. In addition, the strength of the AFt and Gyp corrosion products was much lower than the strength of the concrete matrix.

When the concentration of Na_2_SO_4_ was less than 5%, the corrosion products in concrete were mainly AFt and Gyp crystals, and the volume of the Gyp crystals increased by about 1.24 times [48,49]. Corrosion products generally grow in the direction of lower lateral pressure—i.e., in concrete pores, cracks, and interface transition areas. As the concentration of Na_2_SO_4_ increased, corrosion products gradually accumulated and were stored in the pores and cracks. The large pores in the concrete gradually change to mesopores, resulting in a significant decrease in the proportion of large pores in the concrete and a significant increase in the proportion of mesopores in the concrete. At the same time, more Gyp and AFt crystals were found in pores and cracks. At the same time, the expansion stress and water-absorbing expansion caused by the deposition of AFt and Gyp [50] exceeded the connection force between the pores, thus causing cracks in the matrix structure to crack (generating new or expanded cracks and unicom porein the concrete), and the small pores in the concrete to gradually transfer to the mesopores. Additionally, the Na_2_SO_4_ solution consumed a large amount of Ca^2+^, causing the C-S-H crystals to change from a block structure to a honeycomb shape, which reduced the compactness and compression resistance of the concrete. At the same time, the pores and cracks were filled with a large amount of corrosion products. The proportion of internal small and medium pores was relatively high. Meanwhile, although the internal pore volume ratio of the concrete is reduced to a certain extent, the compressive strength of the corrosion products Gyp and AFt is lower than the strength of the matrix as a whole, making the mechanical properties of the concrete lower than the Q sample as a whole. Therefore, the deterioration rate of the mechanical properties of concrete in a low-concentration environment was relatively small.

When the concentration of Na_2_SO_4_ was greater than 5%, a significant number of Gyp and AFt crystals were produced inside the concrete. The volume expansion of Gyp was larger than that of AFt, and Na_2_SO_4_ crystals also precipitated. The expansion stress generated at this time was much greater than the tensile strength of the matrix, which created new cracks and caused the original cracks in the concrete wider. Besides, a large number of connected pores appear, resulting in intersecting cracks inside the concrete. Further analysis revealed that the corrosion products Gyp and AFt cannot completely fill the internal pores and cracks of the concrete, so that the small and medium pores in the concrete gradually shift to the large pores, and the proportion of the equivalent diameter pores greater than ≥4000 μm increases significantly. In addition, there is a significant increase in the internal mortar and aggregates. The bond strength of the interface transition zone is reduced, which leads to the acceleration of the concrete mechanical properties deterioration.

## 5. Conclusions

This study revealed the damaging potential of different concentrations of Na_2_SO_4_ on the mechanical properties of early-age cast-in-situ concrete from multiple perspectives. The main conclusions obtained in this work can be summarized as follows:

Under a standard curing environment, the compressive strength of Na_2_SO_4_-containing concrete was lower than that of the control sample, and the mechanical properties of concrete gradually decreased with increasing Na_2_SO_4_ concentrations.

The deterioration of concrete by Na_2_SO_4_ was mainly due to the impacts of corrosion products on pores and cracks, which changed the internal microstructure of the concrete and reduced the integrity of the matrix.

As the concentration of Na_2_SO_4_ in concrete increased, C-S-H structures changed from blocks to honeycombs, thus reducing the strength of the concrete matrix. Simultaneously, the proportion of small and medium pores first increased and then decreased, while the proportion of large pores first decreased and then increased with increasing Na_2_SO_4_ concentration. This result reflected the degree of damage that Na_2_SO_4_ can have on the microstructure of concrete.

During the hardening process of cast-in-situ concrete, Na_2_SO_4_ first promoted and then hindered the hydration rate of cement, and Na_2_SO_4_ was unfavorable in terms of the early strength development of the cement. Therefore, Na_2_SO_4_ contamination in cast-in-situ concrete should be strictly prevented.

The research conclusions of this article can make up for the gap in the study of the influence mechanism of different concentrations of Na_2_SO_4_ solution on the early-age mechanical properties, cement hydration rate, pores, and microstructure of cast-in-situ concrete, and it can also help engineers deal with saline soil and salt lake, which provide a reliable reference basis for cast-in-place concrete structures in the region.

## Figures and Tables

**Table 1:** Chemical composition of cementitious materials.

Chemical Composition	Al_2_O_3_	SiO_2_	SO_3_	CL	TiO_2_	Fe_2_O_3_	Na_2_O	K_2_O	MgO	CaO
Content (%)	5.08	20.1	2.02	0.028	0.341	2.94	0.700	0.350	1.50	60.7

**Table 2:** Mix ratio of sodium sulphate concrete.

Tested Specimen	Mixed Sulphate	Sodium Sulfate (kg/m^3^)	Water (kg/m^3^)	Cement (kg/m^3^)	Sand (kg/m^3^)	Gravel (kg/m^3^)
Q	0% Na_2_SO_4_	0	195	402.16	631	1199
3N	3% Na_2_SO_4_	5.85	195	402.16	631	1199
5N	5% Na_2_SO_4_	9.75	195	402.16	631	1199
10N	10% Na_2_SO_4_	19.5	195	402.16	631	1199

**Table 3:** Pore ratio of concrete with different sodium sulfate content on the 28th day.

Designation	Q	3N	5N	10N
Volume Fraction	0.267656	0.224316	0.179699	0.182005
mask volume	9.738 × 10^14^	9.919 × 10^14^	9.28 × 10^14^	9.737 × 10^14^

## Data Availability

Data sharing not applicable.
